# Late ANC initiation and factors associated with sub-optimal uptake of sulphadoxine-pyrimethamine in pregnancy: a preliminary study in Cape Coast Metropolis, Ghana

**DOI:** 10.1186/s12884-021-03582-2

**Published:** 2021-02-02

**Authors:** Benjamin Kwasi Amoako, Francis Anto

**Affiliations:** 1 School of Public Health, University of Ghana, Legon, Accra, Ghana; 2 Cape Coast Metropolitan Health Directorate, Cape Coast, Ghana

**Keywords:** Malaria, Pregnant woman, Sulphadoxine-Pyrimethamine, IPTp-SP, Antenatal care

## Abstract

**Background:**

Malaria infection during pregnancy is of public health importance as it poses risk to the pregnant woman, her foetus and the newborn child. Intermittent preventive treatment during pregnancy using sulphadoxine-pyrimethamine is one way of reducing the effect of the disease on pregnancy outcomes. The study determined factors associated with uptake of sulphadoxine-pyrimethamine during pregnancy in the Cape Coast Metropolis of Ghana.

**Methods:**

A health facility-based cross-sectional study involving pregnant women of ≥36 weeks gestation visiting antenatal clinics in three selected health facilities in the Cape Coast Metropolis was conducted. Participants were consecutively recruited using a structured questionnaire over a 6-week period from May to June, 2018. Descriptive statistics was used to summarize the data whilst Pearson’s chi-square/Fisher exact test was performed to determine associations and logistic regression done to determine the strength of the associations.

**Results:**

A total of 212 pregnant women participated in the study. Formal education, initiating ANC early, taking first dose of SP during second trimester, not experiencing side effects of SP, having knowledge about schedule for taking SP and making ≥4 ANC visits were factors associated with uptake of ≥3 doses of IPTp-SP. Logistic regression analysis revealed that, mothers who made ≥4 ANC visits were 53.77 times more likely to take ≥3 doses of SP compared with those who made < 4 ANC visits (*p* <  0.001). Those who initiated ANC during the first trimester were 3.60 times more likely to receive ≥5 doses compared with those who initiated ANC during the second or third trimester (*p* = 0.022). Making ≥8 ANC visits did not increase the chances of taking ≥5 doses of SP.

**Conclusion:**

Health promotion programmes targeting mothers with no formal education could increase their awareness about the importance of ANC services including uptake of IPTp-SP.

**Supplementary Information:**

The online version contains supplementary material available at 10.1186/s12884-021-03582-2.

## Background

Malaria is a parasitic disease of public health importance transmitted to humans through the bites of infected female *Anopheles* mosquitoes. There are currently five known species of the malaria parasite that cause disease in humans viz.: *Plasmodium falciparum, P. malariae, P. vivax, P. ovale* and *P. knowlesi.* The most severe form of the disease is caused by *P. falciparum* which is the dominant species in Africa [1].

Although malaria is preventable and curable, estimates for 2017 show that 219 million people suffered from the disease resulting in 435,000 deaths [1]. Children under 5 years of age and pregnant women in sub-Sahara Africa suffer most from malaria. In Ghana, the disease is hyper endemic and among pregnant women, it accounts for 17.6% of Out-Patients attendance, 13.7% of admissions and 3.4% of maternal deaths [2].

Currently, intermittent preventive treatment of malaria in pregnancy using Sulphadoxine-Pyrimethamine (IPTp-SP), long-lasting insecticidal nets (LLINs), prompt diagnosis and effective treatment of the disease using artemisinin-based combination drugs are the mainstay of malaria prevention and control in pregnancy [3].

IPTp-SP entails a full therapeutic course of antimalarial medicine given to pregnant women at routine antenatal care visits (ANC). The optimum use of this intervention has been shown to reduce the incidence of pre-term delivery, low birth weight babies [4], maternal anaemia and placental parasitaemia [5].

The current WHO recommendations for IPTp-SP use, require that all pregnant women in areas with moderate to high malaria transmission in Africa should receive at least three doses of SP during each pregnancy. SP administration is to start early in the second trimester, with doses given at least 1 month apart [6]. The national policy of Ghana also requires that SP should be administered to all eligible pregnant women during ANC visits starting from 16 weeks gestation (after quickening), and repeated monthly until the fifth dose is taken between week 32 till delivery [7].

Available data from the Cape Coast Metropolis in the Central Region of Ghana show very low levels of uptake of five doses of SP as recommended by the Ghana Malaria Control Programme since the implementation of the recommendations in 2014 (0.2% in 2014; 5.1% in 2015 and 11.0% in 2016) [8]. The trend however, shows slight regular improvements. Our earlier studies in the Upper East and Greater Accra Regions of Ghana also reported low uptake of five doses of SP [4, 9, 10]. This study in the Cape Coast Metropolis therefore sought to determine factors associated with uptake of IPTp-SP in the Metropolis that could be exploited to improve uptake of the intervention.

## Methods

### Study area and population

The study was carried out in the Cape Coast metropolis located in the Central Region of Ghana. The metropolis covers an area of 122 km^2^ with an estimated population of 182,236 as projected from the 2010 population and housing census. The estimated number of expected pregnancies was 7289, with women in their reproductive age being 43,737 in 2018. The metropolis has been divided into five sub-metropolitan areas for health administrative purposes namely: Adisadel, RCH Central, Ewim, UCC and Efutu [8].

The five sub-metropolitan areas have a total of 105 communities. There are 24 government and 11 private health facilities in the metropolis. The government facilities consist of three hospitals, one polyclinic, three health centres, three clinics and 14 community-based health planning and services (CHPS) compounds. Nine of these government facilities provide both ANC and postnatal care (PNC) services. Three of the government facilities that provide both ANC and PNC services (Cape Coast Metropolitan Hospital, UCC Hospital and Ewim Polyclinic) were randomly selected based on high attendance and range of maternal health services provided [8]. The study population was pregnant women at term (≥ 36 weeks gestation) receiving antenatal care services at the selected facilities.

### Study design

The study was a health facility-based cross-sectional one involving pregnant women of 36 weeks or more gestation attending antenatal clinics in selected health facilities in the Cape Coast Metropolis of Ghana. Participants were sequentially recruited on daily basis into the study over a six-week period as they reported for care during the period May to June, 2018. Thus, on each day of data collection, the first mother to arrive for ANC was contacted, and then the second, the third, in that consecutive order. A questionnaire was administered to the women and data on their socio-demographic characteristics collected. Their antenatal record books were also reviewed and data including gestational age at registration, parity, number of ANC visits, SP uptake and haemoglobin concentration at enrollment extracted.

### Sample size estimation and sampling

The sample size for the study was estimated using the formula n = z^2^p(1-p)/d^2^ by Naing, Winn, & Rusli [11]. Where, z = standard normal deviate of 1.96 (at 95% confidence level), d = the margin of error of (5%) and *p* = the proportion of pregnant women who received IPTp5 = 14.5%, during the first half of the year 2018 [9]. Adjusting for 10% non-response, a sample size of 210 was deemed adequate to detect any significant differences in the factors investigated.

The three selected health facilities (UCC hospital, Cape Coast Metropolitan Hospital and Ewim Polyclinic), in 2016 had a total of 316 pregnant women receiving IPT5. The UCC Hospital had 17 women (5.4%), the Cape Coast Metropolitan Hospital had 127 women (40.2%) and Ewim Polyclinic had 172 women (54.4%). The sample of 210 was therefore distributed proportionately among the three health facilities. A total of 11 women were therefore targeted as a minimum sample for UCC Hospital, 85 for the Cape Coast Metropolitan Hospital and 114 for the Ewim Polyclinic.

Pregnant women who met the inclusion criteria were invited to participate in the study as they arrived at the ANC and those who gave consent to be part of the study were enrolled. Data were collected from these women using a structured pre-tested questionnaire developed specifically for this study. Participants were sequentially recruited into the study as they arrived at the facility on daily basis over a 6-week period until the minimum predetermined sample size was obtained.

### Inclusion/exclusion criteria

All pregnant women of ≥36 weeks gestation visiting the antenatal clinics of the three selected health facilities who gave informed consent (and assent, in the case of those aged 15–17 years) were eligible to participate in the study. This group of women at this stage of pregnancy should be able to have received five doses of IPTp-SP if they received the first dose at gestational age of 16 weeks and continued according to schedule. Pregnant women who had Glucose-6-phosphate dehydrogenase (G6PD) deficiency and those who were referred to the facility for delivery were excluded from the study. This was verified by reviewing the ANC record books for documented G6PD test results and place of enrollment.

### Data collection tool and procedure

A structured questionnaire was developed specifically for this study and used to capture data collected directly from the mothers as well as data extracted from their ANC record books.

Data on the socio-demographic characteristics of the pregnant women, gestational age at registration for ANC, parity, number of ANC visits, SP uptake and any side effects experienced were collected directly from the mothers. Data on compliance with the DOT policy, knowledge of the importance of IPT-SP as well as other facility-related factors were also collected. The ANC record books of the women were reviewed and data on total ANC attendance and number of SP doses received confirmed. Haemoglobin concentration at enrollment was also extracted from the ANC book.

A few more probing questions were asked participants who did not initiate ANC during the first trimester to ascertain reasons for the late initiation. They were prompted to respond ‘yes’ or ‘no’ to seven possible reasons why they could not initiate ANC during the first trimester.

### Quality control

Three research assistants were recruited and trained over a period of 3 days to ensure they understood very well the data collection process. Data collected were reviewed daily for completeness and accuracy. The data collection tool was pre-tested in the Elmina Health Centre located in the Komenda Edina Eguafo Abrem district, which was not part of the study area but with similar structural and functional characteristics. The pre-test was conducted over a period of 2 days, using 25 pregnant women of gestation age ≥ 36 weeks attending ANC. The outcome of the pre-test helped in finalizing the data collection instrument. The research assistants were proficient in English language as well as the local languages (Fante and Twi) mostly used in the study area.

### Data processing and statistical analysis

The collected data were coded and entered into Microsoft Excel, checked for completeness and accuracy and exported to Stata version 15.0 software for analyses. Descriptive statistics such as frequencies, means, ranges and standard deviation were used to summarize the data. During data analysis, the uptake of IPTp-SP was categorized into four groups, < 3 doses or ≥ 3 and < 5 doses or ≥ 5 doses to reflect the WHO and Ghana Malaria Control Programme recommendations respectively. The number of ANC visits was also categorized into four groups, < 4 visits or ≥ 4 and < 8 visits or ≥ 8 visits based on WHO and Ghana Malaria Control Programme recommendations respectively. Chi-square/Fisher Exact test was done to establish association between uptake of SP and each independent categorical variable. Any association with *p*-value < 0.05 was considered statistically significant. Logistic regression analysis reporting odds ratios was used to determine the strength of association between uptake of IPTp-SP and any significant independent variable that was found after the chi-square test.

## Results

### Socio-demographic characteristics of study participants

A total of 212 pregnant women at term (≥36 weeks gestational age), mean gestational age 37.5 weeks (SD: 1.4; range: 36–41), aged 15–44 years (median: 28 years; IQR: 23–32) participated in the study. A total of 12 women were recruited from the UCC Hospital, 85 from the Cape Coast Metropolitan Hospital and 115 from the Ewim Polyclinic. One hundred and six (61.8%) of the women were aged 20–29 years, married (79%) and with at least basic level education (189/212, 89.1%). One hundred and forty-seven (69.3%) of them were in some form of employment with 79% residing in the Cape Coast Metropolis (Table 1).

**Table 1:** Background and obstetric characteristics of participants and IPTp-SP uptake

Characteristics	n	%
**Age group (years)**		
15–19	25	11.8
20–29	106	50.0
30–39	75	35.4
40–49	6	2.8
**Marital status**		
Married	168	79.3
Not married	44	20.7
**Educational status**		
No formal education	23	10.9
Had formal education	189	89.1
**Residence**		
Cape Coast Metropolis	168	79.3
Outside Cape Coast Metropolis	44	20.7
**Occupation**		
Employed	147	69.3
Unemployed	65	30.7
**Parity**		
Nulliparous	52	24.5
Primiparous	72	34.0
Multiparous	88	41.5
**Gestational age at first ANC**		
First trimester	91	42.9
Second trimester	109	51.4
Third trimester	12	5.7
**Gestational age at first dose of SP**		
Second trimester	194	91.5
Third trimester	18	8.5
**Number of ANC visits**		
< 4	24	11.3
≥ 4	188	88.7
< 8	161	75.9
≥ 8	51	24.1
**SP taken under DOT**		
DOT	206	97.2
Not DOT	6	2.8

### Obstetric characteristics of participants, IPTp-SP uptake and heamoglobin concentration at time of initiating ANC

Eighty-eight of the mothers (41.5%) had delivered two or more children, whilst 52 (24.5%) had not delivered before. One hundred and nine (51.4%) of the participants had their first ANC visit in their second trimester, with a few (5.7%) initiating ANC during the third trimester. The number of ANC visits made ranged from 1 to 10 (mean: 6.1, SD: 1.9), with 137 (64.6%) making 4–7 ANC visits and 24.1% making ≥8 visits. The mean gestational age at first ANC visit was 14.2 weeks (range: 4–38, SD: 6.0) (Table 1).

**Fig. 1:**
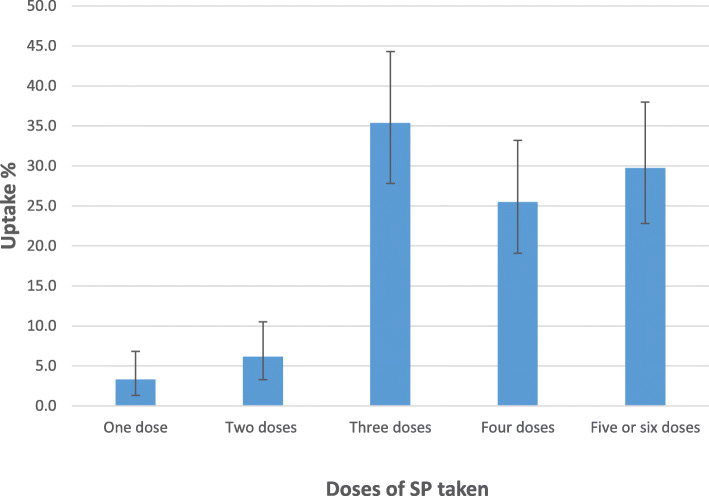
Uptake of IPTp-SP among pregnant women at term in Cape Coast Metropolis, Ghana. The points plotted (−) indicate the percentage of mothers who took the particular number of dose (s) of SP, while the vertical lines show the corresponding 95% confidence intervals

Most of the women (91.5%) received their first dose of SP during the second trimester, with 97.2% of them taking the drug under Directly Observed Therapy (DOT) (Table 1). All the women received at least one dose of SP, with 90.6% taking ≥3 doses and 29.7% receiving ≥5 doses (Fig. 1 (Table 1).

### Reasons given for late initiation of ANC

**Fig. 2:**
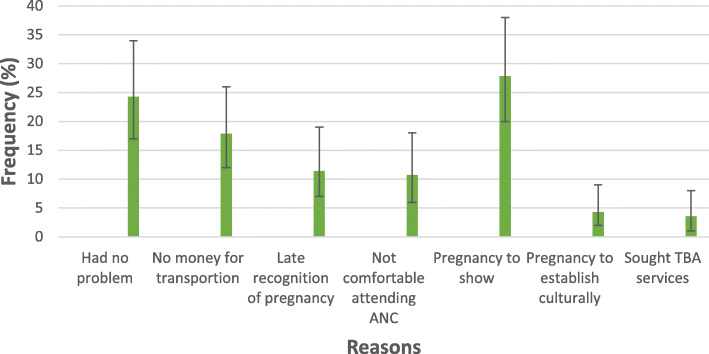
Reasons for late initiation of ANC. The points plotted (−) indicate the percentage of mothers who gave the particular reason for late initiation of ANC, while the vertical lines show the corresponding 95% confidence intervals

A total of 121 mothers initiated ANC during either the second or third trimester (classified as late). They were to respond ‘yes’ or ‘no’ to possible reasons why they could not initiate ANC during the first trimester (classified as early). The main reasons given for not initiating ANC during the first trimester were: waiting for the pregnancy to be physically obvious (27.9%), having no problem or illness associated with the pregnancy (24.3%), no money to pay for transportation and not realizing early that one was pregnant (Fig. 2).

Several factors including formal education and ANC attendance were found to be associated with IPTp-SP uptake. Women who had formal education were more likely to receive ≥3 doses of SP compared with those without formal education (χ^2^ = 16.522, *p* <  0.001). Taking the first dose during the second trimester and making four or more visits enabled the women to receive three or more doses of SP (*p* <  0.05). Early initiation of ANC also enabled the women to receive ≥3 doses of SP (*p* = 0.001). However, there was no significant difference between women who initiated ANC during the first trimester and those who started during the second trimester in terms of taking ≥3 doses of SP (*p* > 0.05) (Table 2). Knowledge of how SP is taken (knowing the interval between doses) also influenced taking of ≥3 doses. Experiencing side effects of SP did not deter the women from taking three doses of the drug.

**Table 2:** Bivariate analysis of background and obstetric characteristics and uptake of three doses of SP

Characteristics	Frequency	IPTp-SP uptake		Chi-square	***p*** value
	***n*** = 212(%)	< 3 doses ***n*** = 22 (%)	≥3 doses ***n*** = 190 (%)		
**Educational level**					
No formal education	23 (10.8)	8 (34.8)	15 (65.2)	16.522	< 0.001
Formal education	189 (89.2)	14 (7.4)	175 (92.6)		
**Gestational age at first ANC**					
First trimester	91 (42.9)	8 (8.8)	83 (39.6)	13.405	< 0.001
Second trimester	109 (51.4)	9 (8.3)	100 (91.7)		
Third trimester	12 (5.7)	5 (41.7)	7 (58.3)		
**Gestational age at first dose of SP**					
Second trimester	194 (91.5)	16 (8.3)	178 (91.7)	11.14	0.001
Third trimester	18 (8.5)	6 (33.3)	12 (66.7)		
**Side effects of SP**					
Side effects	115 (54.3)	7 (6.1)	108 (93.9)	4.975	0.026
No side effects	97 (45.7)	15 (15.5)	82 (84.5)		
**Reason for IPTp-SP**					
Malaria prevention	171 (80.7)	15 (8.8)	156 (91.2)	2.450	0.117
Do not know	41 (19.3)	7 (17.1)	34 (82.9)		
**Interval between SP doses**					
Monthly	188 (88.7)	16 (8.5)	172 (91.5)	6.222	0.013
Does not know	24 (11.3)	6 (25.0)	18 (75.0)		
**Number of ANC visits**					
≥ 4	188 (66.5)	6 (3.2)	182 (96.8)	92.201	< 0.001
< 4	24 (33.5)	16 (66.7)	8 (33.3)		

These same factors (formal education, gestational age at ANC initiation, knowledge and side effects of SP) were associated with uptake of ≥5 doses of SP. More mothers with formal education received ≥5 doses of SP compared with those without formal education (*p* = 0.019). Those who initiated ANC during the first trimester of pregnancy and those who made ≥4 ANC visits were more able to receive five or more doses of SP (*p* <  0.05) (Table 3). Women who had knowledge that SP is taken at monthly intervals and that it helps to prevent malaria were more likely to take ≥5 doses of the drug (< 0.05) compared with those without that level of knowledge. Mothers who experienced side effects from the drug did not take as many doses as those who did not (χ^2^ = 4.618, *p* = 0.032) (Table 3).

**Table 3:** Bivariate analysis of background and obstetric characteristics and uptake of five doses of SP

Characteristics	Frequency	IPTp-SP uptake		Chi-square	***p*** value
	***n*** = 212(%)	< 5 doses ***n*** = 159 (%)	≥5 doses ***n*** = 53 (%)		
**Educational level**					
No formal education	23 (10.8)	22 (95.6)	1 (4.4)	–	0.019*
Formal education	189 (89.2)	137 (72.5)	52 (27.5)		
**Gestational age at first ANC**					
First trimester	91 (42.9)	55 (60.4)	36 (39.6)		
Second & third trimester	121 (57.1)	104 (85.9)	17 (14.1)	18.028	< 0.001
**Gestational age at first dose of SP**					
Second trimester	194 (91.5)	142 (73.2)	52 (26.8)		
Third trimester	18 (8.5)	17 (94.4)	1 (5.6)	–	0.049*
**Side effects of SP**					
Side effects	115 (54.3)	93 (80.9)	22 (19.1)	4.618	0.032
No side effects	97 (45.7)	66 (68.0)	31 (32.0)		
**Reason for IPTp-SP**					
Malaria prevention	171 (80.7)	123 (71.9)	48 (28.1)		
Do not know	41 (19.3)	36 (87.8)	5 (12.2)	–	0.044*
**Interval between SP doses**					
Monthly	188 (88.7)	136 (72.3)	52 (27.7)		
Does not know	24 (11.3)	23 (95.8)	1 (4.2)	–	0.011*
**Number of ANC visits**					
≥ 4	188 (88.7)	135 (71.8)	53 (28.2)	–	0.001*
< 4	24 (11.3)	24 (100.0)	0 (0.0)		

* = Fishers exact test

### Background characteristics of mothers, ANC service utilization and uptake of IPTp-SP

Having formal education, initiating ANC early, taking the first dose of SP during the second trimester, not experiencing side effects after taking SP, having some knowledge about the scheduled time for taking SP and making four or more ANC visits, are factors that were significantly associated with uptake of three or more doses of SP (Tables 2 & Table 3). Adjusting for all of these factors in a logistic regression analysis however revealed that, making four or more ANC visits was the main factor that enabled the pregnant woman to receive three or more doses of SP. Pregnant women who made four or more ANC visits were 53.77 times more likely to take ≥3 doses of SP compared with those women who made < 4 ANC visits (*p* < 0.001) (Table 4). In terms of being able to receive five or more doses however, the main factor identified was early initiation of ANC (AOR: 3.60 (1.20–10.80) as those who initiated ANC during the first trimester were 3.60 times more likely to receive ≥5 doses compared with those who made their firsts ANC visit during the second or third trimester (*p* = 0.022). The analysis further revealed that; making ≥8 ANC visits did not increase the changes of a pregnant woman taking ≥5 doses of SP (Table 5).

**Table 4:** Crude and adjusted association between background characteristics of mothers, ANC service utilization and uptake of ≥3 doses of SP

Characteristics	IPTp-SP uptake		COR	***p***-value	AOR (95%CI)	***p***-value
	< 3 doses ***n*** = 22(%)	≥3 doses ***n*** = 190(%)	(95%CI)			
**Educational level**						
Formal	14 (7.4)	175 (92.6)	*ref*			
No formal	8 (34.8)	15 (65.2)	6.67 (2.41–18.41)	0.040	1.31 (0.20–8.39)	0.776
**Gestational age at first ANC**						
First trimester	8 (8.8)	83 (39.6)	*ref*			
Second trimester	9 (8.3)	100 (91.7)	1.91 (1.17–3.14)	0.009	0.94 (0.31–2.85)	0.918
Third trimester	5 (41.7)	7 (58.3)	0.95 (0.61–1.50)	0.840		
**Gestational age at first dose of SP**						
Second trimester	16 (8.3)	52 (26.8)	*ref*			
Third trimester	6 (33.3)	12 (66.7)	5.56 (1.84–16.80)	0.002	5.37 (0.51–56.82)	0.162
**Side effects**						
No side effects	15 (15.5)	82 (84.5)	*ref*			
Experienced effects	93 (80.9)	108 (93.9)	0.35 (0.14–0.91)	0.031	1.02 (0.21–5.00)	0.981
**Interval between SP doses**						
Monthly	16 (8.5)	172 (91.5)	*ref*			
Does not know	6 (25.0)	18 (75.0)	3.58 (1.25–10.31)	0.018	0.99 (0.14–6.89)	0.993
**Number of ANC visits**						
< 4	16 (66.7)	8 (33.3)	60.67 (1.25–10.31)	< 0.001	53.77 (7.23–399.62)	< 0.001
≥ 4	6 (3.2)	182 (96.8)	*ref*			

*COR* Crude odds ratio *AOR* Adjusted odds Ratio *95%CI* Confidence interval *ref*. Reference

**Table 5:** Crude and adjusted association between background characteristics of mothers, ANC service utilization and uptake of ≥5 doses of SP

Characteristics	IPTp-SP uptake		COR	***p*** value	AOR (95%CI)	***p*** value
	< 5 doses ***n*** = 159(%)	≥5 doses ***n*** = 53(%)	(95%CI)			
**Educational level**						
Formal	137 (72.5)	52 (27.5)	*ref*			
No formal	22 (95.6)	1 (4.4)	8.35 (1.09–63.53)	0.040	9.37 (0.74–119.29)	0.085
**Gestational age at first ANC**						
1st trimester	55 (60.4)	36 (39.6)	*ref*			
2nd & 3rd trimester	104 (85.9)	17 (14.1)	4.00 (2.06–7.77)	< 0.001	3.60 (1.20–10.80)	0.022
**Gestational age at first dose of SP**						
Second trimester	142 (73.2)	52 (26.8)	*ref*			
Third trimester	17 (94.4)	1 (5.6)	6.2 (0.81–47.95)	0.079	1.92 (0.15–22.36)	0.613
**Side effects**						
No side effects	66 (68.0)	31 (32.0)	*ref*			
Experienced effects	93 (80.9)	22 (19.1)	1.98 (1.07–3.73)	0.033	2.41 (0.79–7.32)	0.121
**Interval between SP doses**						
Monthly	136 (72.3)	52 (27.7)	*ref*			
Does not know	23 (95.8)	1 (4.2)	2.95 (1.07–8.17)	0.036	1.61 (0.07–38.32)	0.768
**Number of ANC visits**						
< 8	123 (76.4)	38 (23.6)	1.34 (0.67–2.73)	0.405	0.92 (0.27–3.07)	0.887
≥ 8	36 (70.6)	15 (29.4)	*ref*			

*COR* Crude odds ratio *AOR* Adjusted odds Ratio, *95%CI* Confidence interval, *ref.* Reference

## Discussion

A cross-sectional descriptive study was conducted in three selected health facilities in the Cape Coast Metropolis of Ghana to identify factors associated with uptake of three-five doses of intermittent preventive treatment using Sulphadoxine-Pyrimethamine during pregnancy (IPTp-SP). Uptake of ≥3 doses of SP (as recommended by the WHO, 2012) was found to be high (90.6%). This level of uptake of IPTp-SP was much higher than reports from many other studies in Africa including the 46.6% from the Chokwe district of Mozambique [12], 30.2% uptake among Malawian women [13], 18.0% from Uganda [14] and the 3.1% from the Cross River state, in Nigeria [15]. This level of uptake was however similar to that reported from Sierra Leone (93.2%) [16].

Uptake of ≥5 doses as recommended by the Ghana Malaria Control Programme was found to be low (29.7%). This level of uptake of five doses was however significantly higher than we earlier reported from some other parts of the country (Navrongo, 16.0 and Accra, 14.5%) [4, 9]. It is even likely that a few more will take one more dose of SP as they have not yet delivered and may make another visit before delivery. There has also been consistent improvement in the uptake of ≥5 doses of SP since the implementation of the NMCP recommendations in 2014 (0.2% in 2014; 5.1% in 2015 and 11.0% in 2016) [8], and now 29.7%. Such improvement might be due to education/counselling given to the mothers by the attending midwives as they go for ANC [10]. Thus, women who had knowledge that SP is taken at monthly intervals and that it helps to prevent malaria took more doses of SP.

All the women had taken at least one dose of SP at the time of the study since they were recruited at ANC, with 97.2% of them taking the drug under directly observed therapy (DOT). The main factors found to be associated with uptake of SP were: early initiation of ANC, making four or more ANC visits before term, taking the first dose of SP during the second trimester, experiencing no side effects of SP, having formal education and knowledge about the scheduled time for taking SP.

Efficient ANC services help to reduce maternal and perinatal morbidity and mortality through detection and treatment of pregnancy-related complications, and identification of women at increased risk of developing complications during labour and delivery. Through ANC services, health education and health promotion are enhanced [17]. The level of utilization of these services is dependent on several individual level factors including place of residence of the woman, her level of education and socio-economic status [18].

Early initiation [19] and high utilization of ANC services are factors that are known to strongly predict IPTp-SP uptake [16]. In our current study, early initiation of ANC was found to significantly influence uptake of three-five doses of SP as early initiation of ANC resulted in one being able to make the recommended minimum of four visits and therefore able to receive more doses of SP as reported by other investigators [4, 10, 13, 16, 20]. Additional ANC visits (≥ 8) however, did not seem to lead to uptake of five or more doses of SP when other factors were adjusted for, as reported earlier from Côte d’Ivoire by Toure and colleagues that high ANC attendance does not guarantee higher IPTp coverage [21].

Ghana was among the first countries to adopt the WHO 2012 policy on IPTp-SP [22]; since then various reports have shown some level of improvement in the uptake of SP. As per WHO, 2012 recommendation, most of the women (91.5%) in the current study received the first dose of IPTp-SP early in the second trimester which enabled them to take more doses (3–5 doses) before getting to term. Our earlier study in Accra [9] and Navrongo [4] also reported high levels of early uptake of SP during the second trimester.

A recent report that assessed the uptake of three doses of IPTp-SP in selected malaria-endemic countries in sub-Saharan Africa (Burkina Faso, Ghana, Mali, Malawi, Kenya, Nigeria, Sierra Leone, and Uganda) revealed that, Ghana had the highest level (60.0%) of uptake [23]. Other reports from different parts of the country have also shown high levels of uptake; 71% by Ibrahim and colleagues [24], from Sunyani in the middle forest belt of the country, 76.4% by Anto and colleagues [4] from Navrongo in the northern Savanah belt and 87.5% by Owusu-Boateng and Anto [9] from the southern coastal Savanah belt. Some other studies have however reported low levels of uptake in certain communities of the country; 21–46% by Oppong, et al. [25] from the Kintampo area in the middle forest belt and 32% by Addai-Mensah, et al. [26] from Kumasi also in the forest belt. Thus, uptake of IPTp-SP is uniformly high across the country.

SP is known to be safe and well tolerated by pregnant women as the side effects (including abdominal discomfort, diarrhea, nausea, vomiting, dizziness and headache) are relatively few and mild [27]. These side effects did not influence the willingness of the women to take ≥3 doses of the drug. Fewer of such women who experienced side effects however, continued to take ≥5 doses. According to Onoka, and colleagues [28], in a study among pregnant women in Nigeria, women do not have many concerns about side effects, as they take drugs that health care providers give them because they believe the drugs must be safe.

Formal and higher education significantly influenced the uptake ≥3 doses of SP in the current study as has been reported in some earlier studies. Education up to the secondary level [14] or tertiary [26] enables the mother acquire knowledge and understanding through reading books and newspaper, listening to radio, and watching TV [18], the effects of malaria in pregnancy. Such mothers better understand the effects of the disease on herself, the foetus and the newborn child and also appreciate the benefits of IPTp-SP [24]. Such women also better understand the adverse effects of placental malaria in terms of low birth weight babies and other birth outcomes [29] and therefore, would like to take the recommended doses of SP to get the full benefits of the programme.

## Conclusion

There has been significant improvement in the uptake of ≥5 doses of SP since the implementation of the NMCP recommendations in 2014 (from 0.2 to 29.7%) over a period of 6 years. All the women who participated in the study took at least one dose of SP at the time of the study; with 97.2% of them taking the drug under DOT. Early initiation of ANC, making ≥4 ANC visits before term, uptake of the first dose of SP during the second trimester, experiencing no side effects of SP, having formal education and knowledge about the scheduled time for taking SP were the main factors associated with uptake of SP. Health promotion programmes that will educate mothers on the need to start ANC early, that also target mothers with no or low level of formal education could help increase their level of awareness about the importance of ANC services including the benefits of uptake of IPTp-SP.

### Limitations of the study

The study had some limitations including the fact that participants were recruited into the study at a time that they had not yet delivered. Thus, some could have taken some more doses of SP by the time of delivery and increased the level of uptake. This notwithstanding, the study provides some important factors including the need for early initiation of ANC, that if addressed can improve the level of uptake of IPTp-SP in Ghana and other malaria endemic countries to help achieve the country specific and WHO targets of IPTp-SP uptake. A qualitative study may help through more light on why some women do not initiate ANC early.

## Supplementary Information

**Additional file 1:.** Data set1.

## Data Availability

All data generated during the current study are included in this published article and its supplementary information file (additional file 1).

## Abbreviations

ANC: Antenatal care
DOT: Directly observed therapy
IPTp: Intermittent preventive treatment of malaria in pregnancy
IPTp-SP: Intermittent preventive treatment of malaria in pregnancy with sulfadoxine-pyrimethamine
NMCP: National malaria control programme
PMI: President’s malaria initiative
PNC: Postnatal clinic
SP: Sulphadoxine-pyeimethamine
